# Determinants of mortality among under-five children admitted with severe acute malnutrition in Addis Ababa, Ethiopia

**DOI:** 10.1186/s12937-021-00750-0

**Published:** 2021-12-20

**Authors:** Zebenay Workneh Bitew, Ermias Getaneh Ayele, Teshager Worku, Animut Alebel, Ayinalem Alemu, Frehiwot Worku, Aman Yesuf

**Affiliations:** 1 St. Paul’s Hospital Millennium Medical College, P.O. Box 1271, Addis Ababa, Ethiopia; 2 College of Health and Medical Sciences, School of Nursing and Midwifery, Haramaya University, Harar, Ethiopia; 3 College of Health Science, Debre Markos University, Debre Markos, Ethiopia; 4 Ethipian Public Health Institute, Addis Ababa, Ethiopia; 5 Millennium Medical College, Department of Public Health, St. Paul’s Hospital, Addis Ababa, Ethiopia

**Keywords:** Wasting, Death, Outcome, Predictors, Associated factors, Infants

## Abstract

**Background:**

Management of severe acute malnutrition (SAM) has been a program priority in Ethiopia, but it remains the leading cause of mortality in under-five children. Hence, this study aimed to identify the incidence density rate of mortality and determinants among under-five children with severe acute malnutrition in St. Paul’s Hospital Millennium Medical College, 2012 to 2019.

**Methods:**

A retrospective cohort study was conducted and data were collected using a structured checklist from 673 charts, of which 610 charts were included in the final analysis. The Kaplan-Meier survival curve with Log-rank test was used to estimate the survival time. Bi-variable and multi-variable Cox proportional hazard regression models were fitted to identify determinants of death. Schoenfeld residuals test was used to check a proportional hazard assumption. Goodness of fit of the final model was checked using Nelson Aalen cumulative hazard function against Cox-Snell residual.

**Results:**

In this study, 61 (10%) children died making the incidence density rate of death 5.6 (95% CI: 4.4, 7.2) per 1000 child-days. Shock (Adjusted Hazard Ratio) [AHR] =3.2; 95% CI: 1.6, 6.3)), IV fluid infusion (AHR = 5.2; 95% CI: 2.4, 10.4), supplementing F100 (AHR = 0.12; 95%CI: 0.06, 0.23) and zinc (AHR = 0.45; 95% CI: 0.22, 0.93) were determinants of death.

**Conclusion:**

The overall proportion of deaths was within the range put forth by the Sphere standard and the national SAM management protocol. Shock and IV fluid infusion increased the hazard of death, whereas F100 & zinc were found to decrease the likelihood death. Children with SAM presented with shock should be handled carefully and IV fluids should be given with precautions.

## Background

Malnutrition is a pathologic condition that includes either under or over nutrition [1]. Acute malnutrition, short term deterioration of the nutritional and health status of children, endangers the survival of under-five children [2]. Severe acute malnutrition (SAM) is diagnosed when weight for height is below − 3 z scores of the median World Health Organization (WHO) growth standards or presence of bilateral edema or mid upper arm circumference (MUAC) < 115 mm for a child ≥6 months of age [3]. Globally, one out of three under-five children doesn’t grow well due to malnutrition. An estimated 50 million children are wasted [4], of which the majority (95%) are found in Asia and Africa including Ethiopia [5]. Under nutrition contributes to nearly 45% deaths of under-five children, and the burden is relatively high in low and middle income countries [6]. An estimated 19 million under-five children suffered from SAM and it is estimated to account for approximately 400,000 child deaths each year [7].

Globally, the case fatality rate of SAM has been decreased from 16 to 8% following the implementation of WHO protocols. However, it remains a public health problem in Africa [8]. This could be attributed to high burden of contributing factors (gender, poor socioeconomic status, low birth weight, and residence) in this continent, mainly in sub-Saharan Africa [9, 10]. A cohort study in Kenya substantiates that the coincidence of comorbidities like diarrheal diseases, HIV and pneumonia increases the inpatient and post discharge mortality of children with SAM [11]. The inappropriate implementation SAM management protocols may also have an effect [12]. Ethiopia is placed at the second place in high burden of malnutrition among sub-Saharan countries [13]. This is substantiated by the facts that in Ethiopia, factors like anemia, skin dermatosis, heart failure, poor adherence to management protocols, and impaired vital signs were subsequently reported as contributors of death among children with SAM [14–17].

According to the 2019 Ethiopian Mini Demographic and Health Survey, 7 % of under five children were wasted, of which 1 % were severely wasted [18]. The previous studies conducted in Ethiopia revealed that the prevalence of death among children with SAM ranged from 2.1% in Harar to 28.67% in Waghemra [9, 14–17, 19–29]. This implies that the issue is still a public health problem even though the country has been implementing global and national commitments since 2009 to end all forms of malnutrition by 2030 [30]. The presence of high burden of deaths associated with SAM placed the country as one of the slow progressing countries to address Sustainable Development Goals [31, 32]. The study area is one of the tertiary hospitals in Ethiopia where children referred from all parts of the country are treated for SAM. The data regarding the determinants of mortality in children with SAM is limited in the current study setting, Addis Ababa. This study, therefore, determined the incidence density rate of death and determinants of the mortality among under-five children with SAM in St. Paul’s Hospital Millennium Medical College (SPHMMC), Addis Ababa, Ethiopia.

## Materials and methods

### Study design, study area and period

An institutional based retrospective cohort study was conducted in SPHMMC from October 29/2012 to June 10/2019. Chart review was performed from May 20/2019 to June 20/2019. The college is found at the capital of the country, Addis Ababa. The hospital is one of the very few tertiary governmental hospitals in the country. It has more than 2800 clinical, academic, and administrative and support staff that provide medical services to patients who are referred from all over the country. An average of 250 children with SAM is treated per annum in pediatric ward. Children admitted to the hospital usually receive both clinical and nutritional management services based on the national nutrition protocols. The inpatient management of SAM is offered for free in the hospital. Food supplements are also given for discharged patients for free. In this hospital, children are admitted to the inpatient unit if they have medical complication and either of the followings: weight for height < 70% or weight for height Z-score < − 3, MUAC< 115 mm, bilateral pitting edema or presence of visible severe wasting among children under 6 months of age. Digital weight scales, non-stretchable meters are used to measure weight and length/height of children, respectively. The nutritional status of all children is screened in pediatric outpatient department of SPHMMC.

### Population

The source populations were all the under-five children with SAM admitted to SPHMMC and the study population were all under-five children with SAM admitted to a stabilization center during the study period (from October 29, 2012 to June 10, 2019).

### Inclusion and exclusion criteria

All records of under-five children with SAM admitted to the stabilization center (SC) of SPHMMC from October 29/2012 to June 10/2019 were included in the study. Children with the unrecorded treatment outcome, unknown admission date and unknown discharge date were excluded from the current study.

### Sample size determination

Frist, the sample size was calculated based on the double population proportion formula using Epi Info™ 7 with the assumptions: 95% CI, 80% power ratio of unexposed to exposed 2, outcome in exposed = 15.88% outcome in unexposed 7.85% [24] and risk ratio of 2, and the sample size was 558. The other sample size was computed considering the following statistical assumptions: two-sided significant level (α) of 5%, power 80%, Za/2 = Z value at 95% confidence interval = 1.96, death rate = 28.67%, hazard ratio (HR) = 1.53 [22], which was the hazard death among children who didn’t took vitamin A supplementation. This sample was calculated for Cox proportional hazards model using SATA (version 15). The formula was:

$$N=\frac{E\left(\alpha, \kern0.5em \beta, \kern0.5em \psi \right)}{p_E\left\{S(t),\kern0.5em L(t),\kern0.5em R,\kern0.5em T\right\}}$$

Where:-.

*N* = Sample size

E = is the number of events required to be observed in a study

PE = is the probability of observing an event in a study = 0.2867 [22]

α = Level of significance with 95% confidence interval = 0.05

β = type-II error = 20%

1- Β = 80% = the probability of rejecting type-II error

Ψ = effect size, which is expressed as the log of the hazard ratio (lnHR)

S (t) = Survival Time

L (t) = loss to follow-up (withdrawal)

R = accrual period/ period during which subjects are being enrolled into a study

T = duration of a study

By considering all of the independent determinant of mortality from the reference study all possible sample sizes were computed. The maximum sample (*N* = 673) was obtained by considering the minimum HR, which was the risk of dying among children who didn’t take vitamin A supplement. Therefore, 673 was the final sample size of this study. Finally, 63 samples were excluded based on the exclusion criteria and 610 study subjects were included in the final analysis.

### Data collection procedure

First, documents with SAM were identified from the health information management system (HMIS) register using medical record numbers (MRNs). Accordingly, a total of 1000 eligible documents were selected from the HMIS register. Then, MRNs entered to excel and then to SPPSS version 21 to select the final sample. Finally, 673 samples were selected by computer based generation of random numbers. Cards were reviewed after collecting them from the card room based on the selected registration numbers. A data extraction tool was prepared from the national treatment protocol for the management of SAM [32], SAM registration booklet/HMIS register, SAM multi-chart and by reviewing articles [14, 19, 24]. The data extraction format consisted of socio-demographic data (age, sex, residence), anthropometric measurements (height, weight, MUAC, edema), co-morbidities, types of SAM (marasmus, kwashiorkor or marasmic-kwashiorkor), feeding phase and types of feeding (F75 or F100), frequency of feeding and amount per feed, immunization status, admission & discharge date, referral address as well as medication given and outcomes of the treatment. Six BSc (Bachelor of Science) nurses and two MSc (Masters of Science in nursing) holders were recruited for data collection and supervision, respectively. One day training was given about the tool and the data collectors were deployed to the data collection accordingly. The primary investigator of the study and the supervisors followed the data collection process critically.

### Variables

#### Dependent variable

Time to death from admission to discharge secondary to SAM

#### Independent variables

- Socio-demographic variables (age, sex, address, admission status, status, admission month).
- Co-morbidities (diarrheal disease, dehydration, HIV/AIDS, pneumonia, Protein energy malnutrition (Kwashiorkor/Marasmus), tuberculosis, anemia/pale conjunctivitis, kuashdermatosis, heart failure, shock, meningitis, hypoglycemia, hypothermia, impaired consciousness, congenital heart disease, vomiting and acute kidney injury)
- Vital signs (RR [respiratory rate], PR [pulse rate], SPO2 [oxygen saturation], T^0^[body temperature]))
- Treatments given (immunization, antibiotics, micronutrients, de-worming, NG tube feeding, IV fluids, transfusion and therapeutic foods given like F75, F100, plumpy nut)
- Micronutrient supplements (folic acid, vitamin A, zinc, iron)
- Others like EBF status, bottle feeding, white blood cell level, and hypoglycemia

### Data processing and analysis

Data were coded, cleaned, and entered using Epi Info™ 7 and then exported to STATA version 15 (STATA Corporation, College Station Texas) software for analysis. The presence of missing values, possible outliers, and multicollinearity were checked through exploratory analysis. During the analysis, death was considered as a failure variable and all others were considered as censored observations. The incidence of death with respect to person time at risk was calculated and compared for exposed and unexposed groups. Kaplan-Meier survival curve with log-rank test was fitted to check the presence of a difference in the incidence of death among the categorical variables. Study subjects were followed in days from admission to discharge. Person-days were calculated and the incidence was computed. Both bi-variable and multi-variable Cox regression analyses were done. Finally, variables with *P* ≤ 0.25 in bi-variable Cox regression were selected for the multi-variable Cox regression analysis. Besides, the proportional hazard assumption was also checked using the Log-Log plot and variables fulfilling the assumption were included in the multivariable Cox regression. Unsteadiness of parameter estimate among variables in the final fitted model was checked using variance inflation factor (VIF) and all the individual scores were less than 1.5 with the mean score of 1.18. The Cox regression model for its fulfillment of the proportional hazard assumptions was checked by Schoenfeld residuals test (the global test). The Schoenfeld residuals test confirmed that the assumption was met (*p*-value = 0.48). Finally, model fitness was checked and it can be concluded that the final model fits the data well. The association was summarized using adjusted hazard ratio and statistical significances were tested at 95% CI. Model equation was written as follows:

$$\mathbf{H}\kern0.5em \left(\mathbf{t}\right)\kern0.5em =\kern0.5em \mathbf{ho}\kern0.5em \left(\mathbf{t}\right)\kern0.5em {\mathbf{e}}^{\kern0.5em \left(\mathbf{3.15}\left[\mathbf{Shock}\right]+\mathbf{5}\left[\mathbf{IV}\ \mathbf{fluid}\ \mathbf{infusion}\right]+\mathbf{0.12}\left[\mathbf{F}\mathbf{100}\right]+\mathbf{0.45}\left[\mathbf{zinc}\ \mathbf{supplementation}\right]\right)}$$

Where, H (t) = Hazard rate at time t, ho (t) = baseline hazard at time zero (subject with 0 covariates).

**Fig. 1:**
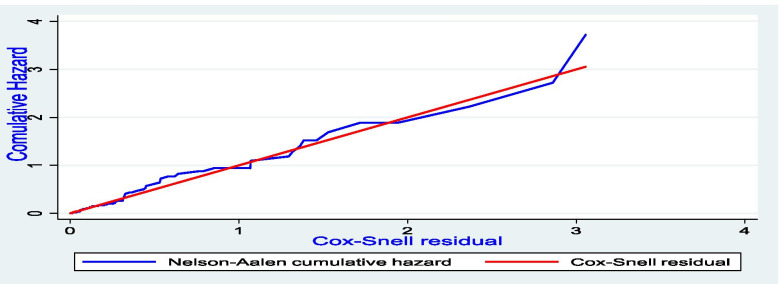
Assessment of model fitness using Cox-Snell residual test to identify the incidence and determinants of death among SAM children at SPHMMC, Addis Ababa, Ethiopia, 2019

The goodness of fit of the final model was also checked using Nelson Aalen cumulative hazard function against Cox-Snell residual. The predict command was used to generate the Cox-Snell residuals from the model. At the end, the Nelson-Aalen cumulative hazard function and the variable can compare the hazard function of the diagonal line. It is shown in the graph that the hazard function follows the 45-degree line very closely over time implying that the model is fitted in this study (Fig. 1).

### Data quality assurance

Data quality was assured by applying properly designed and pre-tested data collection tool. The tool was pre-tested using 5 % of the sample size 1 week before the actual data collection period. This was done to increase its ability to elicit relevant information, to check completeness and consistency. Then, corrective measures were taken accordingly. In addition, training was given for data collectors and supervisor and proper categorization, coding of the questions was made. Finally, data collectors were closely followed by the supervisor and the principal investigator. The completeness and accuracy of the data were closely followed and the primary investigator cross-checked the patient cards for possible data errors.

## Results

### Socio-demographic and admission characteristics

Of the 673 samples, 610 (91%) were included in the final analysis since some records were inappropriately recorded and others were lost from the card room. A total of 610 SAM children was followed for a median of 15 (IQR: 10, 23) days. Of the total study participants, 312 (51.1%) were females and nearly two third 437 (71.6%) came out of Addis Ababa. Most of, 531 (87%) study subjects were new admissions with the mean age of 17 ± 12 months. About half, 323 (53%) of study participants were admitted in the dry season (December to May) (Table 1).

**Table 1:** Socio-demographic and admission characteristics of children with SAM admitted in SPHMMC from 2012 to 2019, Addis Ababa, Ethiopia (*n* = 610)

Characteristics	Treatment Outcome		Total n (%)
	Censored n (%)	Death n (%)	
Age			
< 24 months	423 (90)	47 (10)	470 (100)
≥ 24 months	126 (90)	14 (10)	140 (100)
Sex			
Female	269 (90.3)	29 (9.7)	298 (100)
Male	280 (89.7)	32 (10.3)	312 (100)
Admission			
New	482 (90.8)	49 (9.2)	531 (100)
Readmission	67 (84.8)	12 (15.2)	79 (100)
Residence			
Addis	160 (92.5)	13 (7.5)	173 (100)
Out of Addis	389 (89)	48 (11)	437 (100)
Admission season			
Wet season	251(87.5)	36 (12.5)	287 (100)
Dry season	298(92.3)	25 (7.7)	323(100)
Type of SAM			
Edematous	120 (87.6)	17 (12.4)	137 (100)
Non -Edematous	429 (90.7)	44 (9.3)	473 (100)
Appetite test			
Passed	32 (97)	1 (3)	33 (100)
Failed	138 (85.2)	24 (14.8)	162 (100)
Unknown	379 (91.3)	36 (8.7)	415 (100)
Bottle feeding			
Yes	199 (85.4)	34(14.6)	233 (100)
No	350 (92.8)	27 (7.2)	377 (100)
EBF			
Yes	330 (93.6)	22 (6.4)	352 (100)
No	219 (84.9)	39 (11.1)	258 (100)

*SAM* Severe acute malnutrition, *EBF* Exclusive breast feeding

### Clinical profile and co-morbidity patterns

**Fig. 2:**
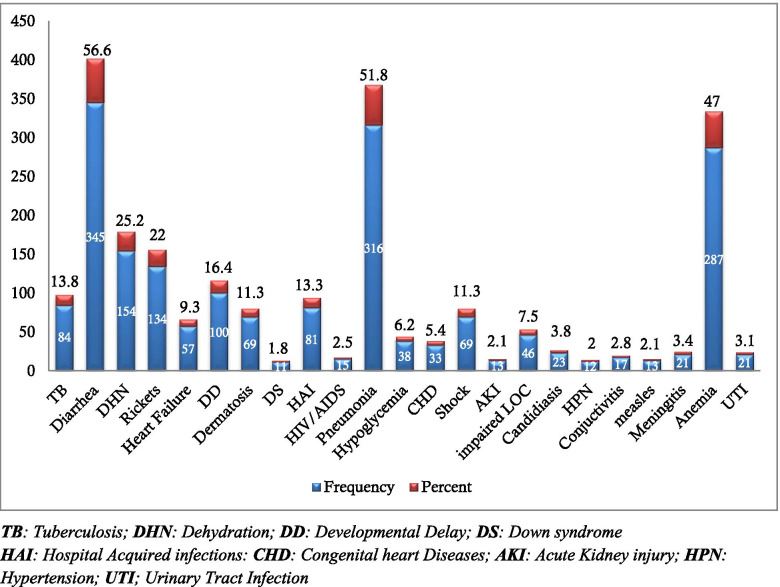
Co-morbidities of SAM children at SPHHMC from 2012 to 2019, Addis Ababa, Ethiopia, 2019

Majority, 345 (56.6%) of the study subjects had diarrheal diseases of which 154 (25.2%) were dehydrated. Pneumonia (51.8%) and anemia (47%) were the second and third common co-morbidities, respectively. The very rare co-morbidities include hypokalemia (2.8%), hyponatremia (1.1%), hypocalcemia (1.3%), gastro-esophageal reflux disease (GERD) (1%), Guillain-Barre syndrome (GBS) (0.5%), hypothyroidism (0.5%), and infantile hypertrophic pyloric stenosis (IHPS) (0.5%) (Fig. 2).

Significant number of children had impaired vital signs during admission. Of the total study subjects, 316 (51.6%), 203 (33.2%), and 121 (19.8%) had altered respiratory rate (bradypnea or tachypnea), altered body temperature (hypo or hyperthermia) and altered pulse rate (bradycardia or tachycardia) during admission. Three quarters, 460 (75.4%) of study participants had decreased oxygen saturation during admission, whereas 24.6% of study subjects had normal (SPO2 > 94%) oxygen saturation in atmospheric air. Regarding immunization status, 263 (43.11%), 134 (22), 132 (21.6%), 81 (13.3%) were fully vaccinated, defaulted, up-to-date and unvaccinated, respectively.

### Treatments given for study participants

Among the study subjects who died, the majority (33.6%) were given IV antibiotics followed by blood transfusion and supplementation of ReSoMal solution accounting 29.5 and 13.4%, respectively (Table 2).

**Table 2:** Treatments given for under-five children with SAM admitted in SPHMMC from 2012 to 2019, Addis Ababa, Ethiopia (*n* = 610)

Variables	Treatment Outcome		Total, n (%)
	Censored, n (%)	Death, n (%)	
IV antibiotics			
Yes	511 (89.5)	60 (10.5)	571 (100)
No	38 (97.4)	1 (2.6)	39 (100)
PO antibiotics			
Yes	175 (96.7)	6 (3.3)	181 (100)
No	374 (87.2)	55 (12.8)	429 (100)
IV fluids			
Yes	93 (66.4)	47 (33.6)	140 (100)
No	456 (97)	14 (3)	470 (100)
Blood Transfusion			
Yes	43 (70.5)	18 (29.5)	61 (100)
No	506 (92.2)	43 (7.8)	549 (100)
ReSoMal			
Yes	304 (86.6)	47 (13.4)	351 (100)
No	245 (94.6)	14 (5.4)	259 (100)
F75			
Yes	458 (89.3)	55 (10.7)	513 (100)
No	91 (93.8)	6 (5.2)	97 (100)
F100			
Yes	474 (95.6)	22 (4.4)	496 (100)
No	75 (65.8)	39 (34.2)	114 (100)
Plumpy nut			
Yes	219 (98.2)	4 (1.8)	223 (100)
No	330 (85.3)	57 (14.7)	387 (100)
Vitamin A			
Yes	241 (94.5)	14 (5.5)	255 (100)
No	308 (86.8)	47 (13.2)	355 (100)
Folic Acid			
Yes	241 (94.5)	14 (5.5)	365 (100)
No	308 (86.8)	47 (13.2)	245 (100)
Zinc			
Yes	183 (93.8)	12 (6.1)	195 (100)
No	366 (88.2)	49 (11.8)	415 (100)
Iron			
Yes	178 (97.8)	4 (2.2)	182 (100)
No	371 (86.7)	57 (13.3)	428 (100)
Vitamin D			
Yes	49 (90.7)	5 (9.3)	54 (100)
No	500 (89.9)	56 (10.1)	556 (100)
De-worming			
Yes	100 (99)	1 (1)	101 (100)
No	449 (88.2)	60 (11.8)	509 (100)
NG tube feeding			
Yes	453 (88.6)	58 (11.4)	511 (100)
No	96 (97)	3 (3)	99 (100)

*PO* Per-mouth, *IV* Intravenous, *ReSoMal* Rehydration solution for malnutrition, *NG* Nasogastric tube

### Incidence of mortality among SAM children

**Fig. 3:**
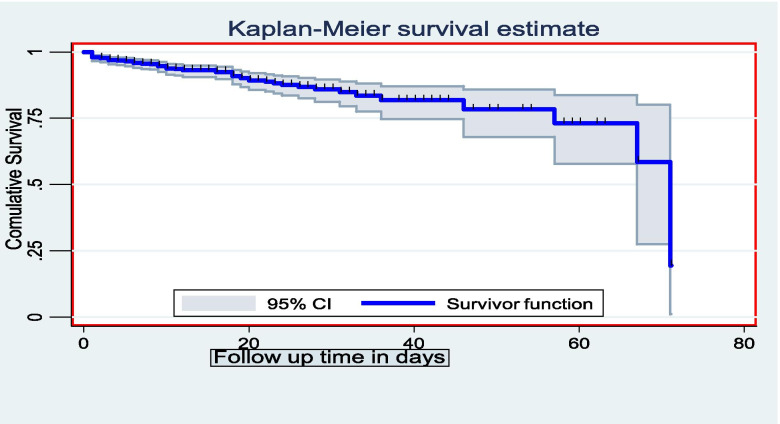
Overall Kaplan-Meier estimation of the survival of admitted SAM children in SPHMMC from 2012 to 2019, Addis Ababa, Ethiopia

The study subjects were followed for a minimum of 1 day and a maximum of 71 days giving a total of 10,829 person-days of observation, total follow up time of all study subjects. Out of the 610 study subjects, 61 (10%) died at the end of the follow up and the overall incidence density rate (IDR) of death was 5.6 (95% CI: 4.4, 7.2) per 1000 child-days or 2.05 per child-year. The highest incidence death rate was recorded in the first 2 days making the IDR 11.8 per 1000 person days (95% CI: 6.99, 19.9). The IDR of death at the end of 7th day and 14th day were 6.55 per 1000 person-days (95% CI: 4.5, 9.6) and 5.5 per 1000 person-days (95% CI: 4.0, 7.5). A total of 48 children died in the first 20 days of follow up. The cumulative probabilities of survival in the 5th, 10th, and 20th days were 96.4, 94, and 89%, respectively. The mean survival time of study participants was 59 days (95% CI: 55.8, 62.60) (Fig. 3).

**Fig. 4:**
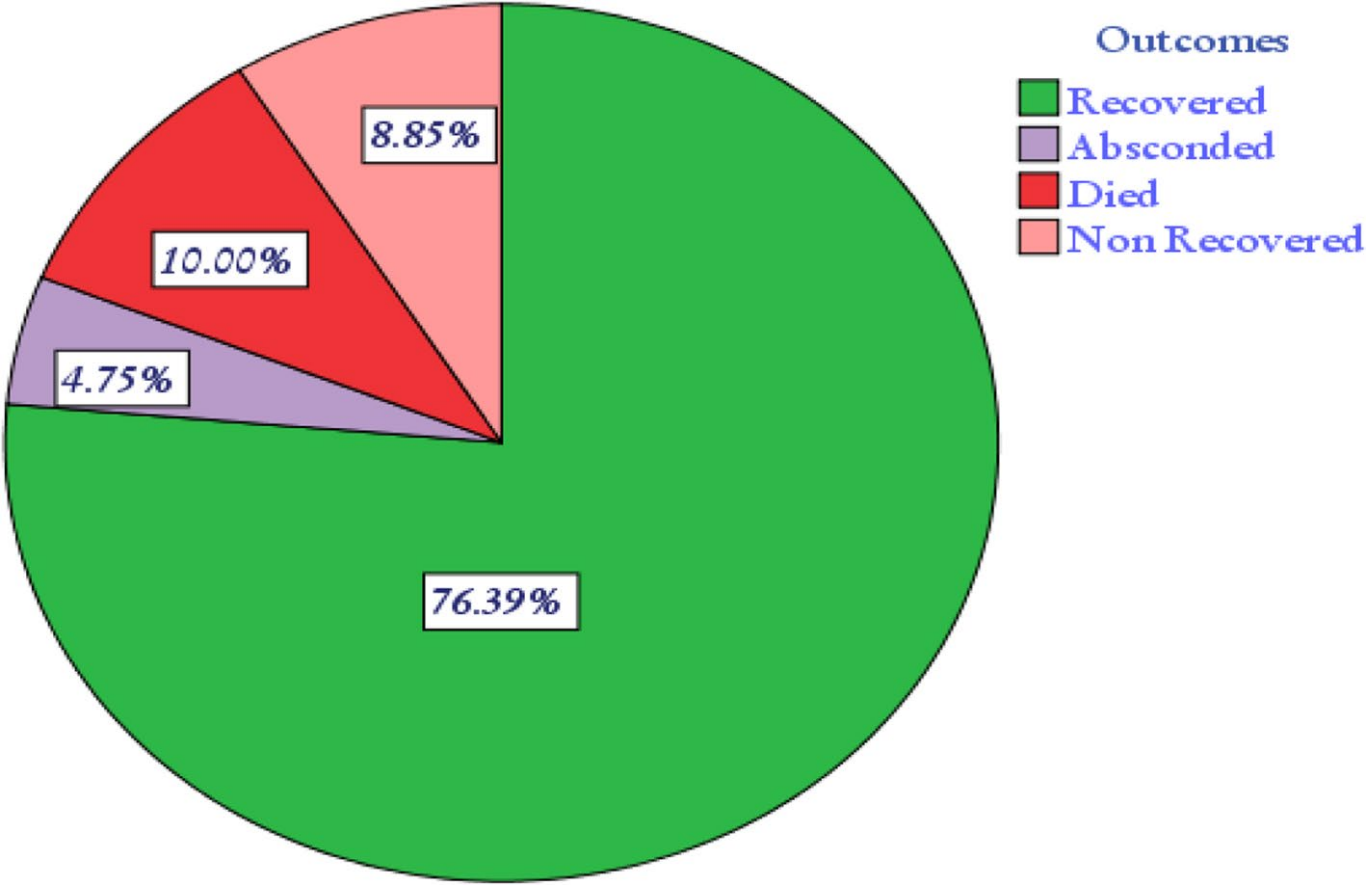
Treatment outcome of SAM children in SPHMMC from 2012 to 2019, Addis Ababa, Ethiopia

**Fig. 5:**
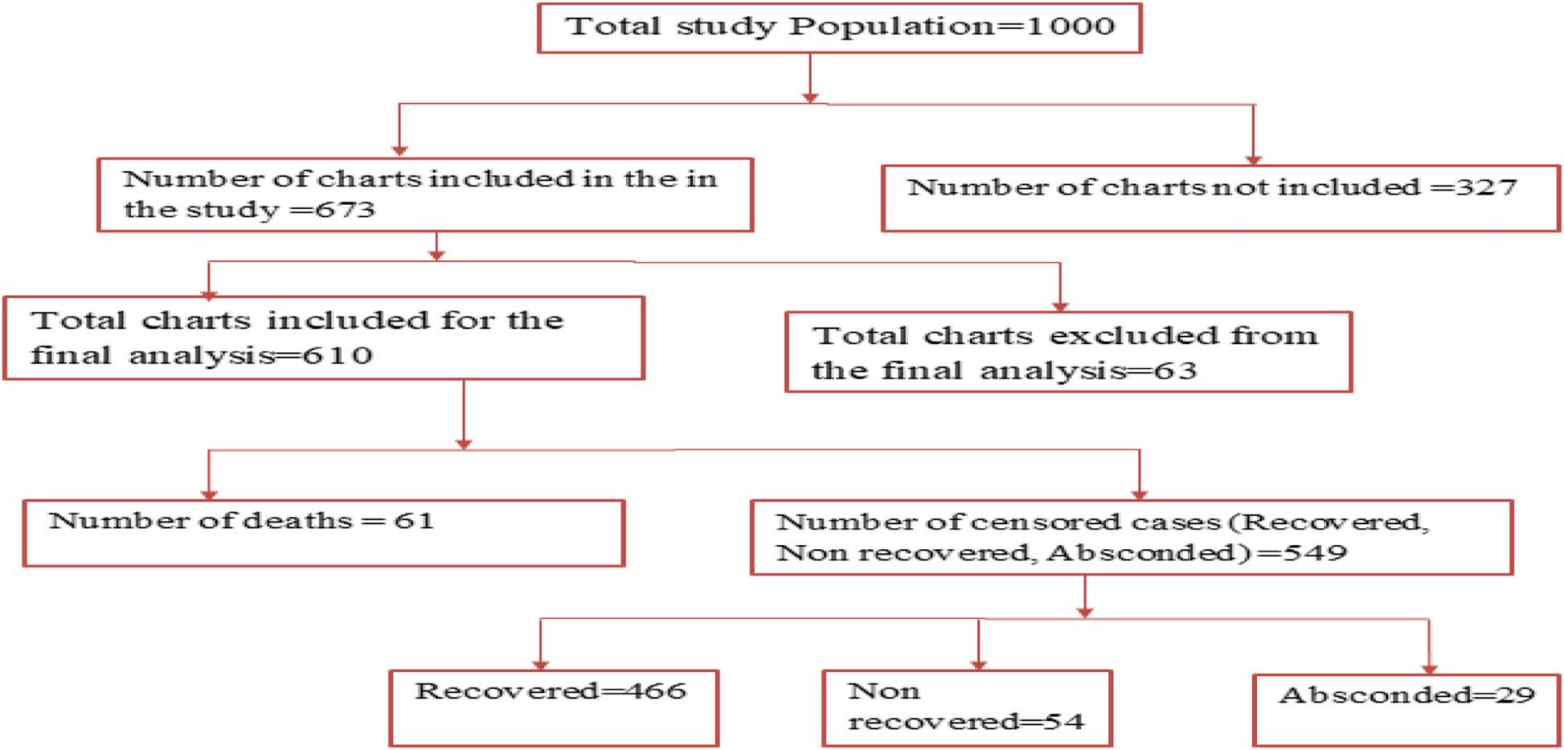
Chart Schematic presentation of study participants’ recruitment and allocation process

Of the total study subjects, 549 (90%) were censored of which the majority, 466 (74.6%) recovered at the end of the follow up; 54 (8.9%) and 29 (4.8%) of participants non-recovered and absconded, respectively (Figs. 4 and 5).

The IDR of selected variables was calculated and incidence among the exposed and the non-exposed groups were calculated as shown in the following table (Table 3). The overall IDR of death was 5.6 per 1000 child-days. The highest IDR of death was found to be related to shock (26.8) and least was due to iron (1.1).

**Table 3:** Incidence density rate of death stratified by the determinant variables among severely malnourished under five children admitted to SC in SPHMMC, 2012 – 2019

Variables	Frequency	Person-day	Death	IDR (95% CI)
Overall	610	10,829	61	5.6 (4.4–7.2)
Respiratory Rate				
Normal	294	5001	21	4.2 (2.7–6.4)
Altered	316	5828	40	6.8 (5–9.4)
HIV/AIDS				
Yes	15	229	4	17.5 (6.6–46.5)
No	595	10,600	57	5.4 (4.1–6.9)
Pneumonia				
Yes	316	5633	39	6.9 (5–9.5)
No	294	5196	22	4.2 (2.8–6.4)
Hypoglycemia				
Yes	38	600	13	2.2 (1.3–3.7)
No	572	10,229	48	4.7 (3.5–6.2)
CHD				
Yes	33	586	7	11.9 (5.6–25)
No	577	10,243	54	5.3 (4–6.9)
Shock				
Yes	69	1118	30	26.8 (18.8–38.4)
No	541	9711	31	3.2(2.2–4.5)
AKI				
Yes	13	267	5	5.3 (4–6.9)
No	597	10,562	56	18.7 (7.8–45)
LOC				
Normal	564	9810	45	4.6 (3.4–6)
Impaired	46	1019	16	15.7 (9.6–25.6)
F100				
Yes	496	9515	22	2.3 (1.3–3.5)
No	114	1314	39	29.7 (21.7–40.7)
Vitamin A				
Yes	255	4450	14	3.1 (1.9–5.3)
No	355	6379	47	7.4 (5.5–9.8)
Folic Acid				
Yes	365	6528	23	3.5 (2.3–5.3)
No	245	4301	38	8.8 (6.4–12)
Zinc				
Yes	195	3777	12	3.2 (1.8–5.6)
No	415	7052	49	6.9 (5.3–9.2)
Iron				
Yes	182	3616	4	1.1 (0.4–2.9)
No	428	7213	57	7.9 (6.1–10.2)
IV fluid				
Yes	140	2615	47	17.9 (13.5–23.9)
No	470	8214	14	1.7 (1–2.8)
ReSoMal				
Yes	351	6364	47	7.4 (5.5–9.8)
No	259	4465	14	3.1 (1.8–5.3)

*CHD* Congenital heart diseases, *AKI* Acute kidney injury, *LOC* Level of consciousness, *ReSoMal* Rehydration solution for malnutrition

### The Kaplan-Meier survival function of the determinant variables

**Fig. 6:**
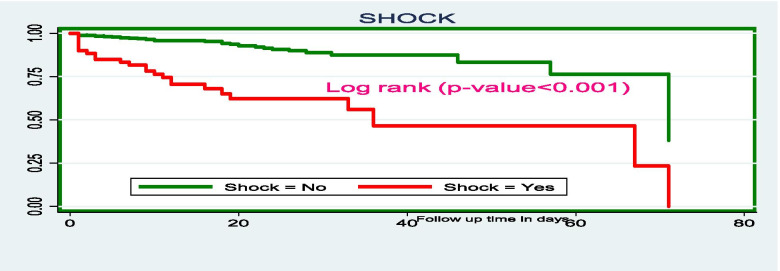
The Kaplan-Meier survival curves comparing the survival time of SAM children with shock at SPHMMC, Addis Ababa, Ethiopia, 2019

The differences in the survival probability of the independent determinants of time to death were computed using the Cochran-Mantel Haenszel Log rank test. The finding of this study pinpointed that those children who had SAM with shock have lower survival time as compared to the counterparts. The mean survival time of SAM children with shock was 37 days (95% CI: 28, 46.5) whereas, the mean survival time for those who hadn’t shock was 63 days (95% CI: 59.5, 6.5) (Fig. 6).

**Fig. 7:**
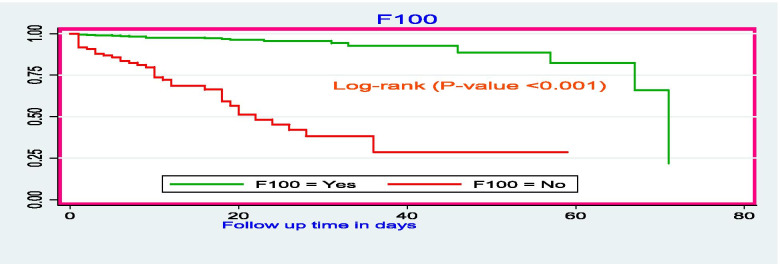
The Kaplan-Meier survival curves comparing the survival time of SAM children who took F100 as nutritional treatment in SPHMMC, Addis Ababa, Ethiopia, 2019

**Fig. 8:**
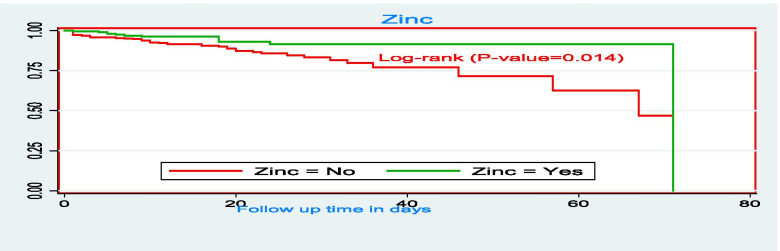
The Kaplan-Meier survival curves comparing the survival time of SAM children who took zinc as part of treatment in SPHMMC, Addis Ababa, Ethiopia, 2019

**Fig. 9:**
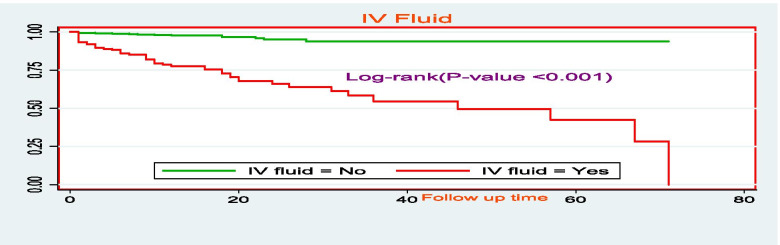
The Kaplan-Meier survival curves comparing the survival time of SAM children who took zinc as part of treatment in SPHMMC, Addis Ababa, Ethiopia, 2019

The survival time for those who took F100 therapeutic food had relatively higher survival time as compared to those who didn’t take F100 therapeutic food with mean survival times of 64.7 days (95% CI: 61, 68 days) and 28.7 days (95% CI: 22, 35 days), respectively (Fig. 7). The mean survival time of SAM children who took zinc had higher (66 days, 95% CI: 63, 69 days) as compared to the counterparts (55.8 days: 95% CI: 51, 60.6 days) (Fig. 8). There was a significant difference in the duration of survival children who were given intravascular fluids with a mean survival time of 43 days (95% CI: 36.6, 49 days). Children who didn’t take IV fluids had higher survival time with the mean survival time was 67.7 days (95% CI: 65.7, 69.6 days) (Fig. 9).

### Bi-variable and multi-variable Cox regression analysis

In the bi-variable Cox regression, the socio-demographic variables (residence & season of admission), variables related to feeding and supplements (bottle feeding, EBF practice, IV fluid, ReSoMal, blood transfusion, F100, vitamin A, folic acid, iron, and zinc), co-morbidities (CHD, shock, HIV/AIDS, pneumonia, hypoglycemia, AKI, LOC, CHF, DHN, meningitis, and HAI), and impaired vital signs (RR & SPO2) were found to be significant at *p*-value ≤.0.25. However, the variables that met the proportional hazard assumption using log-log plot were; IV fluid, ReSomal solution, F100, vitamin A, folic acid, iron, zinc, CHD, shock, HIV/AIDS, pneumonia, hypoglycemia, AKI, LOC and RR were fitted for multivariable Cox regression analysis.

In the multi-variable Cox-regression analysis, shock was found to be the sole co-morbidity determining the death of SAM children. The hazard of death among children who had shock was 3.15 (95% CI: 1.56, 6.34) times higher than those who hadn’t shock. Administration of IV fluids, F100, and zinc were the other independent determinants of time to death in this study. Children treated with IV fluids were five (95% CI: 2.42, 10.4) times at higher risk of death as compared to those who were not given the IV fluids. Children who took F100 as a supplementary treatment were 82% (AHR = 0.12; 95% CI: 0.06, 0.23) less likely to die as compared to the counterparts. Children who were supplemented zinc were 55% (AHR = 0.45; 95% CI: 0.22, 0.93) less likely to die at any given (during the follow up period of this study) time than those who were not provided zinc (Table 4).

**Table 4:** Multivariable Cox regression model showing the distribution of factors associated with death in severely malnourished children admitted to SC in SPHMMC from 2012 to 2019 (*N* = 610)

Independent variables	Treatment outcome		CHR (95% CI)	AHR (95% CI)
	Censored n (%)	Dead n (%)		
IV fluids				
Yes	93 (66.4)	47 (33.6)	10.23 (5.6–18.7)	5 (2.42–10.4)*
No	456 (97)	14 (3)	1	1
ReSoMal				
Yes	304 (86.6)	47 (13.4)	2.34 (1.29–4.25)	1.26 (0.64–2.5)
No	245 (94.6)	14 (5.4)	1	1
F100				
Yes	474 (95.6)	22 (4.4)	0.07 (0.04–0.12)	0.12 (0.06–0.23)*
No	75 (65.8)	39 (34.2)	1	1
Vitamin A				
Yes	241 (94.5)	14 (5.5)	0.44 (0.24–0.9)	0.47 (0.22–0.99)
No	308 (86.8)	47 (13.2)	1	1
Folic Acid				
Yes	241 (94.5)	14 (5.5)	0.41 (0.25–0.7)	1.38 (0.71–2.68)
No	308 (86.8)	47 (13.2)	1	1
Zinc				
Yes	183 (93.8)	12 (6.1)	0.46 (0.25–0.87)	0.45 (0.22–0.93)*
No	366 (88.2)	49 (11.8)	1	1
Iron				
Yes	178 (97.8)	4 (2.2)	0.13 (0.05–0.36)	0.61 (0.2–1.88)
No	371 (86.7)	57 (13.3)	1	1
RR				
Normal	273(92.8)	21 (7.2)	1	1
Altered	276 (87.3)	40 (12.7)	1.69 (0.99–2.88)	1.1 (0.58–2.1)
HIV/AIDS				
Yes	11 (73.3)	4 (26.7)	3.33 (1.2–9.2)	1.54 (0.5–4.66)
No	538 (90.4)	57 (9.6)	1	1
Pneumonia				
Yes	277 (87.6)	39 (12.4)	1.7 (1–2.87)	1.32 (0.73–2.4)
No	272 (92.5)	22 (7.5)	1	1
Hypoglycemia				
Yes	25 (65.8)	13 (34.2)	4.8 (2.6–8.96)	1.28 (0.62–2.68)
No	524 (91.6)	48 (8.4)	1	1
CHD				
Yes	26 (78.8)	7 (21.2)	2.35 (1.06–5.2)	1.72 (0.71–4.2)
No	523 (90.6)	54 (9.4)	1	1
Shock				
Yes	39 (56.5)	30 (43.5)	7.74 (4.65–12.86)	3.15 (1.56–6.34)*
No	510 (94.3)	31 (5.7)	1	1
AKI				
Yes	8 (61.5)	5 (38.5)	3.76 (1.5–9.44)	1.72 (0.62–4.7)
No	541 (90.6)	56 (9.4)	1	1
LOC				
Normal	519 (92)	45 (8)	1	1
Impaired	30 (65.2)	16 (34.8)	3.17 (1.7–5.88)	0.57 (0.26–1.26)

*Significant at *P* < 0.05

*AHR* Adjusted hazard ratio, *IV* Intravenous, *ReSoMal* Rehydration solution for malnutrition, *RR* Respiratory rate, *CHD* Congenital heart diseases, *AKI* Acute kidney injury, *LOC* Level of consciousness

## Discussion

This study intended to determine the incidence and determinants of mortality among under-five children admitted with SAM in the SC of SPHMMC from 2012 to 2019. A total of 610 SAM children were followed for 10,829 children-days and the incidence density rate of death was found to be 5.6 per 1000 child-days. The cumulative probabilities of survival in the 5th, 10th, and 20th days were; 96.4, 94, and 89%, respectively and the mean time of survival was 59 days. The presence of shock, IV fluid infusion, and supplementation of F100 & zinc were independent determinants of death.

At the end of the follow up, 10% of the children with SAM died. This is comparable with the minimum Sphere standard and the national management protocol for severe acute malnutrition managed at stabilization centers (< 10%) [32]. The current finding also is in agreement with the findings of a study performed in Uganda (9.8%) [33]. However, this finding is slightly higher than the studies conducted in several parts of Ethiopia such as in Hadya Zone (7%) [28], Dilchora Hospital (7.6%) [17], Debremarkose (5.9%) [26], Jima Zone (9.3%) [24], Tigray (3.8%) [14], Gedeo Zone (9.3% [16], Southern Ethiopia (9.3%) [23], Felegehiwot Referal Hospital (8.47%) [34], Nekemte Referral Hospital (4.4%) [27], North Shoa Zone Hospitals (5.8%) [35], South Wollo Zone (3.4%) [36] and Hiwot Fana Specialized University Hospital (2.1%) [29]. This finding is also higher than the study results from Sudan, Malawi and India [12, 37, 38]. The difference could be resulted from differences in the demography of study subjects and it might be also associated with delayed referral process of SAM children to the current study area. On the other hand, the proportion of deaths in this study is lower than the findings of the studies done in Dilla University Referral hospital (12.4%), Gondar University Hospital (12.52%), Yirgalem Hospital (16%), Mekele city (12.8%), Hawassa University Comprehensive Specialized Hospital (10.8%) and Sekota Hospital (29%) [15, 19, 22, 25, 39, 40]. These disparities may be attributed to the differences in the level of care given in the current study area and relatively improved diagnostic process of the co-morbidities, which can facilitate prompt care to be given to SAM children to decrease the mortality rate. The studies done in Zambia, Ghana, and Uganda also revealed that 40.5, 13.2, and 11.9%, respectively, of SAM children died, which is significantly higher than the current finding [41–43]. This could be because of the variation in the clinical profile of study subjects during admission and difference in the health care system.

The incidence of death of this study (5.6 per 1000 child-days) is lower than the findings of researches done in North West Ethiopia, Mekele City, and Dilla University Hospital with the incidence density rates of 10.4 deaths per 1000 child-days, 7.3 per 1000 child-days, and 7.57 per 1000 child-days, respectively [15, 19, 39], but higher than the finding of a study conducted in Tigray (3.2 per 1000 child-days) [14]. The possible elucidation for the difference could be because of the variation in the type of care and level expertise of clinicians, severity of co-morbidities and differences in management as well as variability in the organization of the therapeutic feeding units.

Coming to the mean survival time, the mean time of survival for the present study is 59 days. This is relatively lower as compared to the results of studies in North West Ethiopia (56 days) and Gedo Zone (79.6 days) [15, 16], but higher than the results found from the studies conducted in Tigray region (41.93 days) and Dilla University Hospital 47 (days) [14, 19]. The discrepancy could be attributed to variation in the clinical profile of study subjects during admission and the time of admission of study participants at the first admission since children admitted to SPHMMC were referred from the four corners of the country. Thus, it is inevitable for children to have delayed admission to our study area which could affect the mean survival time of children.

In this study, shock was identified as the main determinant of death of under-five children and the hazard of death of children with shock was three times as compared to the counterparts. This is line with the findings prior studies in Ethiopia [15, 16, 19], of which shock was identified as the main determinant of death. This is because of the fact that children with SAM are highly at risk of shock secondary to severe infections and diarrheal diseases which can cause either hypovolemic or septic shocks. Besides, severe sepsis and diarrheal diseases in malnourished children might be associated with low cardiac reserves leading to shock which leads to death [44]. Likewise, the hazard of death among children who were infused IV solution was significantly higher than children who didn’t take IV fluids. This finding is consistent with previous Ethiopian studies done by Adal et al. [19], Fikre et al. [40], Gebremichael et al. [39], and Wangnew et al. [15] who pinpointed that IV infusion fastens the risk of death. This is because of the fact that during severe acute malnutrition, there is a scientific elucidation that during SAM, mainly when there is protein energy malnutrition, the internal vital organ activities may either decrease or shut down as an adaptive response. The subsequent physiological changes like decreased renal and cardiac function, as part of surviving mechanism, could make study participants prone to secondary complications while IV fluid infusion. It is also common to have high intercellular sodium and low potassium secondary to reductive adaptation [45]. Due to these justifications, routine administration IV-fluid is not recommended because it could lead to complications like fluid overload, cerebral edema, heart failure, and finally to death. Currently, it controversial to give fluids because there is poor evidence on resuscitation with IV fluids for SAM children [46]. It is also very challenging to diagnose fluid volume deficit in severely malnourished children which might lead to maladministration of IV fluids.

In the present study, adherences to the nutritional therapies such as F100 and zinc supplementation are found to be protective from death. F100 is found decrease mortality by 82%. This is in agreement with the findings of studies conducted in Southern Ethiopia [40], in North West Ethiopia [15], and in Dilchora Hospital, Eastern Ethiopia [17]. Adherence to the standard nutritional management could enhance early recovery. It is evident that supplementation of F100 in the form of either diluted or undiluted has a pivotal role in restoring the normal physiology of children after reductive adaptation and it could help study participants with SAM to achieve catch up growth and to gain weight [47]. This may be explained further that the nutritional contents of F100 (high calorie, proteins, low fat, and almost all vitamins and minerals) may enhance catch up growth [48]. Supplementations of zinc can decrease the death of SAM children by 55%. In most of the literatures that we reviewed zinc was not reported as a predictor of time to death of SAM children, but it is the main factor in this study. The possible rational could be enlightened by the fact that in children with severe acute malnutrition, micronutrient deficiencies are common. Due to this reason, supplementation of micronutrients in the first 2 weeks as part of SAM management is recommended. Zinc is one of the recommended trace minerals which must be given in this period to enhance the rehabilitation process of SAM children [44]. In addition, SAM is commonly associated with diarrheal diseases and dehydration could lead to zinc depletion [49].

In the current study, diarrheal diseases, anemia, HIV infection, pneumonia and altered vital signs were not significantly associated with the death and this is in contrast with the findings of the other studies which were conducted prior to this study [19, 21, 34, 41, 50]. The difference might be also resulted from differences recording system as well as due to variations in the geospatial distribution of co-morbidities. The other possible rationale may be the fact that the intermediate effect of the treatment given could obscure the true effects of those variables with death. Incompleteness of records and absence confirmatory tests for co-morbidities were limitations of this study. Besides, selection bias might affect the true estimates.

## Conclusion

The overall proportion of deaths was within the range put forth by the Sphere standard and the national SAM management protocol. Besides, this study found that there was a high rate of mortality in the first few days of admission. From the independent determinants of death, shock and infusion of IV solution increased the hazard of death. Supplementation of F100 and zinc were preventive factors of death. Hence, children with SAM must be critically managed in the first few days at the stabilization centers to decrease the mortality rates. In addition, IV fluid infusions should be administered with great precautions. It is also recommended clinical trials should focus about administering zinc for all children with SAM and on the types IV fluids to be given for children with SAM.

## Data Availability

The data sets used and/or analyzed during the current study are available from the corresponding author on reasonable request.
